# Online quantitative monitoring of live cell engineered cartilage growth using diffuse fiber-optic Raman spectroscopy

**DOI:** 10.1016/j.biomaterials.2017.06.015

**Published:** 2017-09

**Authors:** Mads S. Bergholt, Michael B. Albro, Molly M. Stevens

**Affiliations:** a Department of Materials, Imperial College London, London SW7 2AZ, United Kingdom; b Department of Bioengineering, Imperial College London, London SW7 2AZ, United Kingdom; c Institute of Biomedical Engineering, Imperial College London, London SW7 2AZ, United Kingdom

**Keywords:** Fiber-optic Raman spectroscopy, Articular cartilage, Tissue-engineering, Live cell Raman spectroscopy, Online biomedical Raman spectroscopy

## Abstract

Tissue engineering (TE) has the potential to improve the outcome for patients with osteoarthritis (OA). The successful clinical translation of this technique as part of a therapy requires the ability to measure extracellular matrix (ECM) production of engineered tissues *in vitro*, in order to ensure quality control and improve the likelihood of tissue survival upon implantation. Conventional techniques for assessing the ECM content of engineered cartilage, such as biochemical assays and histological staining are inherently destructive. Raman spectroscopy, on the other hand, represents a non-invasive technique for *in situ* biochemical characterization. Here, we outline current roadblocks in translational Raman spectroscopy in TE and introduce a comprehensive workflow designed to non-destructively monitor and quantify ECM biomolecules in large (>3 mm), live cell TE constructs online. Diffuse near-infrared fiber-optic Raman spectra were measured from live cell cartilaginous TE constructs over a 56-day culturing period. We developed a multivariate curve resolution model that enabled quantitative biochemical analysis of the TE constructs. Raman spectroscopy was able to non-invasively quantify the ECM components and showed an excellent correlation with biochemical assays for measurement of collagen (R^2^ = 0.84) and glycosaminoglycans (GAGs) (R^2^ = 0.86). We further demonstrated the robustness of this technique for online prospective analysis of live cell TE constructs. The fiber-optic Raman spectroscopy strategy developed in this work offers the ability to non-destructively monitor construct growth online and can be adapted to a broad range of TE applications in regenerative medicine toward controlled clinical translation.

## 1 Introduction

Cartilage tissue engineering is a promising osteoarthritis (OA) treatment strategy that involves the generation of live replacement tissues that can be used to repair clinical cartilage defects. In a conventional approach, chondrogenic cells are encapsulated in a polymeric scaffold, which provides them with an environment to support the synthesis and elaboration of a new cartilage extracellular matrix (ECM) over time. This technique aims to generate tissues that recapitulate a robust, mechanically-functional cartilage ECM, capable of supporting physiologic mechanical loads. A key goal of cartilage tissue engineering is to implant tissues after they have developed suitable levels of the major cartilage ECM constituents (*i.e.*, glycosaminoglycans (GAGs) and collagen), allowing them to resist mechanical strains, provide a low friction articulating interface and, ultimately, exhibit long term survival in the native environment of the synovial joint. In this context, the field of cartilage tissue engineering has achieved growing success [1], [2]. To date, the development of several culture strategies have allowed for the generation of engineered cartilage with ECM content and mechanical properties that match those present in native articular cartilage [3], [4].

The successful clinical translation of cartilage tissue engineering requires the development of robust techniques for the assessment of tissue quality and levels of ECM content prior to implantation. The development of measurement techniques may be particularly crucial for engineered cartilage derived from human patient cell populations (autologous chondrocytes, mesenchymal stem cells [MSCs], or induced pluripotent stem cells [iPSCs]), which exhibit highly variable growth rates [5]; as such, these tissues require varying *in vitro* culture durations before they have deposited a level of ECM that is suitable for implantation. The premature implantation of tissues can potentially lead to failure upon exposure to the native mechanical loading environment. The ability to perform an online monitoring of tissue growth over time, which can guide the optimal point of tissue implantation, may be essential for achieving future translational success of cartilage tissue engineering strategies.

Conventional techniques for assessing the ECM content of engineered cartilage, such as biochemical assays and histological staining, are inherently destructive. Further, the utilization of these conventional assessment techniques to monitor tissue growth over time would require the parallel fabrication of sacrificial engineered cartilage samples. Given requisite high cell densities needed to achieve sufficient ECM deposition and the inherent challenge of procuring large amounts of cells from patients, this sacrificial strategy is likely to be highly burdensome. Sample sacrificing becomes even more prohibitive when engineering large cartilage tissues for the repair of clinical sized OA defects or for replacing an entire articular cartilage surface.

To date, various techniques have been applied for online monitoring (i.e. nondestructive tissue monitoring during culture) including microdialysis [6], magnetic resonance imaging [7] and micro-computed tomography (microCT) [8]. These modalities are however either invasive, require expensive equipment, and/or do not provide specific molecular information about the tissue engineered constructs. Interestingly, near-infrared (NIR) and mid-infrared (MIR) spectroscopy have been applied to quantify collagen, GAG, and water [9], [10], [11], [12]. These monitoring techniques are associated with several limitations: infrared spectroscopy is associated with limited photon penetration due to water absorption in the wavelength range (>1100 nm) and has inherently less molecular specificity [13]. Alternatively, Raman spectroscopy is a highly promising inelastic light scattering technique that can probe the vibrational modes of molecular bonds in tissue samples. This technique can be used to interrogate the biochemical composition (*i.e.*, biochemical conformation of proteins, carbohydrates, lipids, nucleic acids, etc.) of tissues with high molecular specificity [14]. For instance, Raman spectroscopy has previously been performed on commercially available engineered oral mucosa, scaffolds, and tissue pellets [15], [16], [17], [18]. Raman spectroscopy can be performed in aqueous environments and may hold key advantages over NIR spectroscopy in its high biomolecular specificity and probing of highly hydrated tissues, such as articular cartilage [19], [20]. We have recently demonstrated the ability of 532 nm Raman microspectroscopic imaging to quantify the relative distribution of the major ECM constituents (GAG and collagen) in devitalized tissues [20]. Visible laser excitation, however, poorly penetrates tissue. Since articular cartilage represents a thin avascular tissue layer (∼3 mm thickness) that is highly transparent in the tissue optical window (*i.e.*, 700–1100 nm) [21], it could represent an excellent tissue for diffusely probing the bulk biochemical composition using NIR laser excitation.

Here we develop a novel diffuse fiber-optic Raman spectroscopy strategy and introduce a comprehensive workflow designed to monitor and quantify ECM biomolecules in live cell tissue engineered cartilage constructs online. In this study, we explore the potential utility of this methodology by assessing: 1) the potential deleterious effect of Raman spectral acquisitions on construct viability and growth, 2) the ability of Raman spectral acquisitions to quantify the concentrations of ECM constituents in constructs (via multivariate curve resolution analysis) and quantify the spectral similarity between engineered and native cartilage tissues (via principle component analysis), and 3) the ability of fiber-optic Raman spectroscopy to monitor long term, live tissue growth over a 6 week culture period. The presented strategy could potentially allow us to define tissue specifications to identify engineered cartilage that is suitable for implantation, in order to achieve future translational success of cartilage tissue engineering.

## 2 Materials and methods

### 2.1 Tissue samples: native and engineered cartilage

Immature articular cartilage cylindrical explants were harvested from the femoral condyles of 4-week old calf knee joints obtained from a local abattoir (N = 3 animals). Explants were extracted via biopsy punch and the superficial and deep zones of the tissue were excised, yielding cylindrical explants of predominantly middle zone cartilage (∅4 × 2 mm). Explants were stored dry at −20 °C for up to two weeks until testing. Tissue engineered cartilage constructs were prepared from primary bovine articular chondrocytes obtained from 4-week old bovine calf carpometacarpal joints (N = 5 animals per study). Tissue was enzymatically digested for 15 h in 1.5 mg/mL of type-IV collagenase (Invitrogen) and isolated cells were encapsulated in 2% (w/v) type VII agarose at a nominal density of 30 × 10^6^ cells/mL and configured as ∅4 × 2 mm tissue engineered (TE) construct disks, as described previously [22]. These relatively small constructs were utilized for this investigation to mitigate the introduction of spatial heterogeneities, as supported by histological characterizations in prior investigations on this TE system [23]. Constructs were cultured for up to 56 days in a chondrogenic media formulation, consisting of high glucose DMEM supplemented with 100 nM dexamethasone, 100 μg/mL sodium pyruvate, 50 μg/mL l-proline, 1% ITS + premix (6.25 μg/mL insulin, 6.25 μg/mL human holotransferrin, 6.25 ng/mL selenium), 1% PS/AM antibiotic antimycotic, and 173 μM ascorbate-2-phosphate. Media was supplemented with 10 ng/mL TGF-β3 or maintained TGF-β free.

### 2.2 Mechanical testing and biochemical assays

The equilibrium compressive Young’s modulus of TE constructs was determined using a custom-made testing apparatus consisting of a stainless steel loading platen and an inline load cell (500 g; Honeywell) mounted to a micrometer-actuated vertical translating stage (Newport Spectra Physics). While immersed in phosphate buffered saline (PBS), TE constructs were subjected to a 10% axial compressive strain and allowed to undergo stress relaxation until equilibrium (reached at 20 min). The subsequent reaction force was recorded and used with sample geometry for modulus calculations. A two-way ANOVA was performed (α = 0.05 and statistical significance set at *p* < 0.05) to assess the effect of culture duration and TGF-beta supplementation on the Young’s modulus of TE constructs. A Tukey’s HSD post-hoc test was run to examine differences between the groups.

Following Raman analysis and mechanical testing, TE constructs were analyzed for their biochemical contents. Each construct was digested via proteinase-K (0.5 mg/mL; 56 °C for 16 h) and subsequently processed for its GAG and collagen contents, via the dimethylmethaline blue [24] and orthohydroxyproline [25] assays, respectively.

### 2.3 Live cell Raman spectroscopy instrumentation

**Fig. 1:**
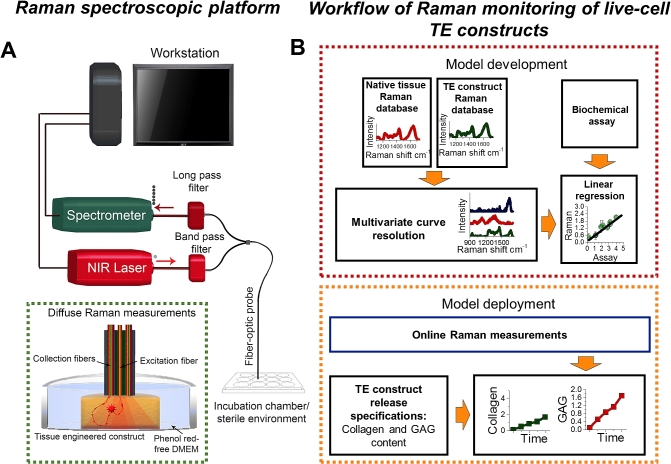
(**A**) Schematic of the fiber-optic Raman spectroscopy system for monitoring and biochemical quantification in live cell tissue-engineered (TE) constructs. (**B**) Workflow of Raman spectroscopic monitoring of live cell TE constructs. Also shown is the developed computational processing framework for online evaluation of TE constructs.

The integrated fiber-optic Raman spectroscopy and culture system capable of live cell spectral acquisition is shown in Fig. 1A. The in-house built Raman system consists of an NIR diode laser (λ_ex_ = 785 nm) (maximum output: 500 mW, B&W TEK Inc.), and a fiber-coupled scientific grade spectrograph (QEPro, Ocean Optics Inc) equipped with a gold-coated, reflective mirror, a gold-coated 1200 grooves/mm grating, and a 64 × 1044 pixel back-thinned thermo electric-cooled NIR-optimized charge-coupled device (CCD) camera (Hamamatsu Inc. Japan). The spectrometer was fiber-coupled, using a linear array of 7 × 100 μm low OH fibers (Thorlabs Inc, Newton, NJ). We further integrated in-line bandpass and longpass filters (LL01-785-25 and LP02-830RU-25, Semrock Inc, USA, NY) to reduce the silica background signals generated from the fiber-optics. The fiber-optic system acquires Raman spectra from articular cartilage and TE constructs with a spectral resolution of ∼8 cm^−1^ in the range 800–1800 cm^−1^ using a 100 μm entrance slit. To realise live cell monitoring in culturing environments we integrated a low-OH RP21 fiber-optic probe (Thorlabs Inc, Newton, NJ) with a culture plate to enable continuous monitoring and sampling of different wells under sterile conditions in incubator environments. The fiber-optic probe (∅3.2 mm) comprises a central 200 μm excitation fiber and 6 × 200 μm collection fibers for bulk diffuse sampling. The fiber-optic system allows for the acquisition of spectral information while tissues are maintained in their culture environment. All native articular cartilage tissues and TE constructs were measured in hydrated conditions. The fiber-optic Raman probe was placed in contact with the native articular cartilage and TE constructs. Each Raman spectrum was measured with an acquisition time of 30 s and one accumulation with 250 mW of 785 nm laser excitation to ensure high signal to noise ratio. Before each measurement, the probe was sterilized with 70% ethanol. The atomic emission lines of argon and mercury spectral lamp (HG-1, Ocean Optics, Inc., Dunedin, FL) were used for wavelength calibration.

### 2.4 Online data processing

To control the entire system, we developed a comprehensive computational framework in C/C++ (Visual Studio 10, Microsoft Inc.) (Fig. 1B). This framework enables efficient data collection and online application of multivariate statistical analysis based on models developed in the PLS-toolbox Matlab 2014b (Mathworks, Natick, MA). The framework interfaces with the spectrometer CCD using the Omnidriver/SPAM development platform (Ocean Optics, Inc., Dundin, FL). All native articular cartilage and TE construct Raman spectra in this work were first subjected to cosmic ray rejection using the first derivative spectrum. If there were no cosmic rays present, it was then corrected for the CCD dark noise and fiber-optic probe background spectrum. To remove the fiber background we applied multivariate curve resolution (MCR) to quantify the fiber background signal. This background signal was then subtracted to yield the Raman/autofluorescence spectrum alone. We further removed the autofluorescence by subtracting a constrained fitted 3rd order polynomial (PLS-toolbox Matlab 2014b (Mathworks, Natick, MA)). The spectra was then subjected to outlier detection using PCA [26]. If the spectrum passed the outlier test, it was fed to the multivariate predictor to enable prospective application for TE monitoring. All the data processing was performed online to enable continuous monitoring of TE constructs. Multivariate statistical models were developed using PLS-toolbox 8.0.2 (Eigenvector Research, Manson, WA) in the Matlab 2014b (Mathworks, Natick, MA) programming environment on a Linux Ubuntu v15.04 multicore server (12 core, i7 3.3 Ghz processors, 64 Gb memory).

### 2.5 Spectral similarity metrics between constructs and native tissue

Raman spectroscopy is able to provide a label-free and exhaustive biomolecular fingerprint that could allow us to define tissue release specifications prior to implantation based on its overall signature. In consideration of the biological variability exhibited by TE constructs, an analysis can be performed to account for underlying spectral variations and quantitatively assess the spectral similarity between TE constructs and native tissue. In this analysis, we applied a multivariate statistical process strategy to evaluate the biomolecular signature of the TE constructs. We developed a principal component (PCA) model based exclusively on native articular cartilage. This model was then applied to the live cell TE constructs over time. Before PCA, mean-centering was applied to remove common variance. Q-residuals and Hotelling T^2^ statistics are two complimentary parameters that were used as similarity metrics to compare the biomolecular variability of TE constructs with that of native tissues [26]. Briefly, Q-residuals provide an assessment on variation not captured by the PCA model. Hence Q-residuals can be used to evaluate how well the TE construct Raman spectra fit the developed native tissue PCA model. Contrary, Hotelling’s T^2^ gives an indication of extreme score values within the PCA model. This value, therefore, provides information about abnormal biochemical variations (e.g., unusual concentrations of collagen, GAG and water in the ECM). Hence, the smaller the values of Q-residuals and Hotelling’s T^2^, the more the TE construct mimics native tissue. To determine if a TE construct spectrally can be considered similar in biomolecular composition to native tissue we utilize the normalized 95% confidence intervals (CIs) of the Q-residuals and Hotelling T^2^ for native tissue as thresholds.

### 2.6 Effect of Raman laser excitation on construct growth

Raman spectral acquisitions generally require the use of high laser excitation intensity to obtain high spectral signal to noise ratios from highly hydrated tissues, such as native and engineered cartilage. Since the intense NIR light could potentially induce thermal effects, we performed an initial test to assess the potential detrimental effect of prolonged high laser excitation on the viability and growth of TE constructs. For this study, freshly cast constructs (n = 5 per group) were subjected to a continuous laser excitation (250 mW; 785 nm) from our fiber-optic probe for either 0, 2, 5, or 15 min under sterile, hydrated conditions. For the laser excitation period, constructs were immersed in an open Petri dish filled with phenol red-free DMEM, while maintained in a sterile tissue culture hood. After laser excitation, all constructs were returned to culture, analyzed for viability (live/dead™ imaging) the following day, and analyzed for GAG and collagen synthesis levels after an additional two weeks of culture.

### 2.7 Raman spectral biochemical quantification of frozen tissue engineered constructs

In order to quantify collagen, GAG, and water in TE constructs, a multivariate model was applied to a dataset of Raman spectra obtained from a population of tissue constructs. MCR assumes that the biologically complex Raman spectrum of cartilage and engineered constructs can be described as a linear combination of a set of pure component spectra weighted according to their abundance in each spectrum. Provided that the spectra dataset reflects sufficient biochemical variation, MCR analysis can extract from the dataset: 1) a pure spectra for each modelled biochemical component, and 2) the relative concentration (in arbitrary units) of each biochemical component in each sample. The utility of this approach was similarly demonstrated in our prior imaging work in quantifying the spatial distribution of ECM in sectioned native and engineered cartilage [20]. Here, an MCR model consisting of three component spectra was applied to the Raman dataset from a population of constructs grown under varying conditions: cultured for 0, 14, 28, 42, and 56 days in the presence or absence of TGF-β (n = 4 per group). At the end of culture, all samples for model development were frozen and stored for subsequent Raman spectroscopic and biochemical analysis. In order to assess the validity of the MCR model, the extracted three pure component spectra were compared to reference chemical spectra of the dominant molecular constituents of engineered cartilage: water (PBS), chondroitin sulphate (Sigma-Aldrich), and type II collagen (Sigma-Aldrich). These chemicals were measured and processed in the same way as the tissue Raman spectra. The successful determination of the pure component spectra, in principle, gives rise to the relative concentration of GAG, collagen, and water in each sample, expressed in arbitrary units. In order to assess the accuracy of these measures, Raman-measured relative concentrations were compared to absolute concentrations, as determined via biochemical assay measurements.

### 2.8 Online monitoring of live cell tissue engineered construct growth

An additional study was performed in order to demonstrate the ability of this fiber-optic Raman probe system to perform long-term online monitoring of the growth of TE cartilage using the developed model. Here, an additional batch of constructs (n = 5) was cultured for up to 42 days in the presence of TGF-β. For each construct, the Raman spectrum was acquired after 0, 14, 28, and 42 days of culture under sterile, hydrated conditions. Raman spectra were obtained via a 30 s acquisition time to ensure a sufficient signal to noise ratio. Raman measurements were performed with constructs situated in phenol red-free DMEM to avoid autofluorescence during Raman spectral acquisitions. Our previously implemented MCR model was applied prospectively to quantify the relative concentration of GAG and collagen in these constructs at each time point.

## 3 Results and discussion

### 3.1 Effect of Raman laser excitation on construct growth

**Fig. 2:**
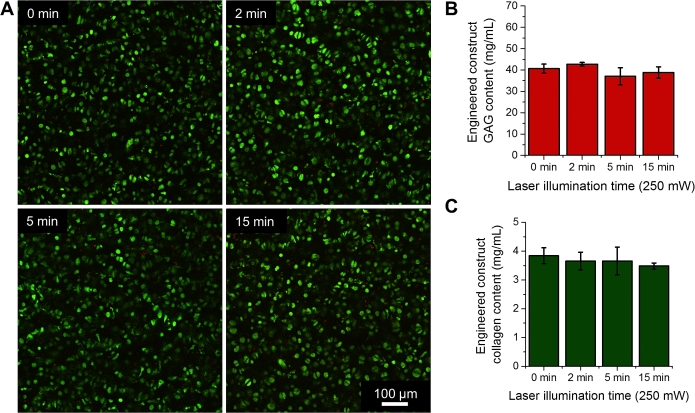
(**A**) Images of live cells (green) and dead cells (red) after an initial exposure to 785 nm laser for continuous 0, 2, 5, or 15 min duration. (**B-C**) GAG and collagen contents of TE constructs 2 weeks after initial exposure to 785 nm laser probe for 0, 2, 5, or 15 min. (For interpretation of the references to colour in this figure legend, the reader is referred to the web version of this article.)

Raman spectroscopy generally requires the use of high laser excitation power to obtain Raman spectra of sufficient signal from highly hydrated tissues. After an initial application of 250 mW, 785 nm laser excitation for up to 15 min, we did not observe any cell death in TE constructs (Fig. 2A) or statistical reduction in ECM synthesis (GAG [*p* = 0.12] or collagen [*p* = 0.56] secretion [Fig. 2B–C]). This is likely because articular cartilage and TE constructs contain no known NIR chromophores and the optical properties are predominantly dominated by diffuse light scattering [21]. Moreover, the high degree of tissue hydration ensures that cooling effects, such as evaporation and perfusion, prevent excessive heating of the articular cartilage and TE constructs. It is important to note that for our live cell online monitoring of tissue growth, the acquisition of sufficient Raman spectral signal required the use of 30 s laser excitation durations. As such, it is highly encouraging that this extreme case of 15 min excitations shows no evidence of disrupting tissue growth.

### 3.2 Raman spectral biochemical quantification model development based on frozen tissue

**Fig. 3:**
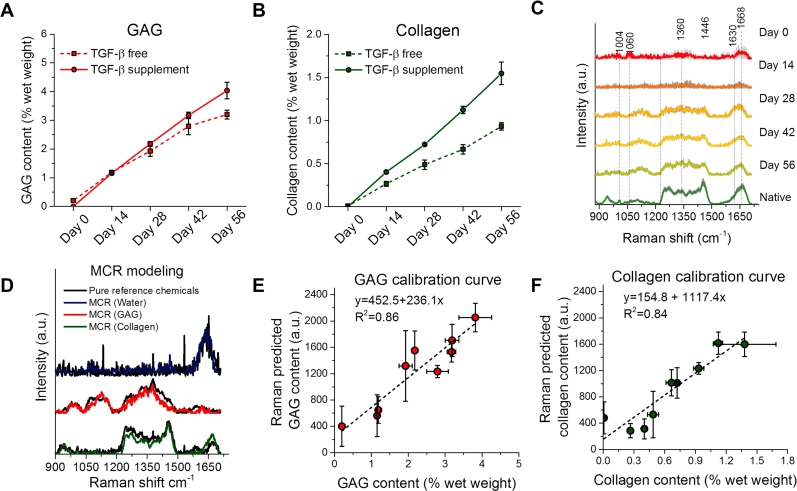
(A–B) Biochemically measured GAG and collagen contents of TE constructs (TGF-β free and TGF-β supplemented) after 0, 14, 28, 42, and 56 days of culture. (C) Mean Raman spectra ± 1 standard deviation (SD) of native cartilage and TE constructs after varying culture durations. (D) Multivariate curve resolution (MCR) developed from native and tissue-engineered (TE) constructs (TGF-β free and TGF-β supplemented). The MCR pure components correlate well with laboratory grade water, GAG, and collagen (water R^2^ = 0.62, GAG: R^2^ = 0.80, and collagen: R^2^ = 0.80) (E–F) Correlations between the Raman prediction (in arbitrary units) and biochemically measured GAG and collagen, respectively, with linear fits to the data. Raman spectroscopy showed an excellent correlation with biochemical assays for measurement of collagen (R^2^ = 0.84) and GAG (R^2^ = 0.86). The correlation for prediction of water content was poor (R^2^ = 0.16) due to inherently weak signals in the range 800–1800 cm^−1^ and is therefore not shown.

According to biochemical assay measurements, TE constructs increased in GAG and collagen contents over time (Fig. 3A–B). Contents increased further with TGF-β supplementation, consistent with prior investigations [27]. We then measured a Raman spectral database of native articular cartilage tissues (N = 3 animals, n = 7 tissues, n = 67 Raman spectra). We performed measurement of cultured TE constructs (n = 75) over a time course of 56 days under the two growth-factor conditions (TGF-β free and TGF-β supplemented) and sacrificed them at various time points (*i.e.*, 0, 14, 28, 42, and 56 days). The average Raman spectra ±1 standard deviation (SD) of native articular cartilage and TE constructs at various time-points showed highly reproducible signatures with subtle biomolecular variability (Fig. 3C). Raman peaks previously identified in articular cartilage were found near 1061 ν_s_(S=O) of GAG, 1245 cm^−1^ (amide III *ν*(C-N) and δ(N-H) of collagen), 1450 cm^−1^ (δ(CH_2_) of collagen), 1630 cm^−1^ (H_2_O) and 1668 cm^−1^ (Amide I *ν*(C=O) of collagen) [20], [28], [29]. These mean Raman spectra reveal that the biomolecular compositions of the TE constructs gradually become more similar to native articular cartilage over time, as ECM deposition accumulates. Further, the results at day 0 are dominated by water signals at 1630 cm^−1^. Hence, the agarose scaffold does not interfere significantly with the tissue Raman spectra.

To quantify the content of collagen and GAG, we developed a three-component MCR model. Fig. 3D shows the MCR pure components that correspond to water (PBS), GAG, and collagen. These MCR components are in exceptional agreement with Raman spectra of respective pure laboratory grade biochemicals (collagen: R^2^ = 0.80, GAG: R^2^ = 0.80 and water R^2^ = 0.62) [20]. The small discrepancy between the Raman spectra of the pure laboratory grade chemicals and those presented in our previous publication [20] can be attributed to subtle artifacts originating from the silica of the fiber-optics in the current implemented Raman probe system. These results prove that the MCR model is specific to the respective biochemical components, and, as such, allows for the faithful extraction of the relative concentration of GAG and collagen in each TE construct sample.

To assess the accuracy of these Raman ECM measurements, we correlated these results with the concentration of GAG and collagen in each TE construct, as independently assessed via biochemical assays. These results show a remarkable linear relationship between Raman spectroscopy and biochemical assays for collagen (R^2^ = 0.84) and GAG (R^2^ = 0.86) (Fig. 3E–F). It is intriguing that the changing optical properties of TE constructs over time does not prevent biochemical quantification due to changing depth of penetration and collection efficiency of the excitation and scattered Raman photons. Hence, our results indicate that the diffuse fiber-optic Raman sampling approach is exceptionally robust against varying optical properties during tissue growth in the NIR spectral range.

It is important to note that the output of the Raman based MCR analysis provides only the concentration of GAG and collagen in arbitrary units. The calibration curves (as shown in Fig. 3E–F) can be used to convert the Raman measured ECM concentrations to absolute concentrations. As such, these calibration curves can be readily developed for specific engineered tissue systems and used in conjunction with fiber-optic Raman spectra, allowing for a measure of the absolute concentration of ECM constituents in TE constructs.

In this work we initially applied the standard supervised multivariate technique (*i.e.*, partial least squares (PLS) analysis). In PLS analysis, biochemical reference concentrations are used together with cross-validation to guide the regression. We, however, found that while PLS can provide excellent predictions, the quantification is deceitful. This is because the collagen and GAG content covariates over time during tissue culturing (R^2^ = 0.92). Hence, we must emphasise that much care should be taken in the interpretation of PLS results when modelling such temporal datasets and extracting information about individual molecular constituents. Here we showed that by using MCR as an unsupervised and unbiased approach, we could extract estimates of pure ECM components that correlate well with reference biochemicals. This, together with validation against biochemical assays, represents direct evidence that our model is both highly specific and sensitive to the respective ECM components.

**Fig. 4:**
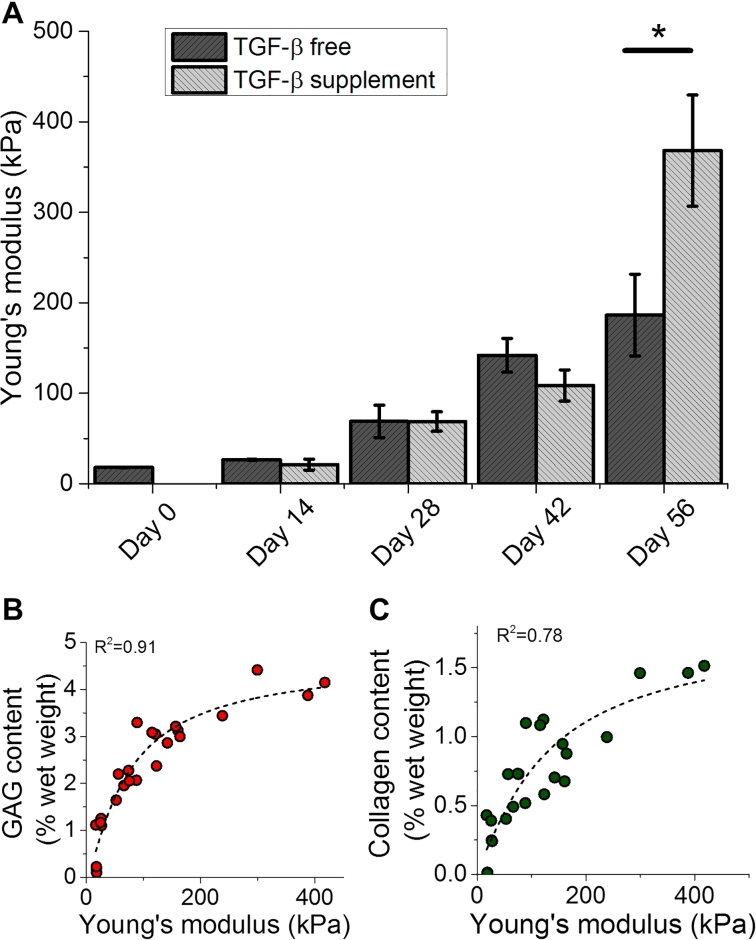
(**A**) Mechanical properties (compressive Young’s modulus) of TE constructs (TGF-β free and TGF-β supplemented) at various time points (*i.e*. 0, 14, 28, 42, and 56 days). *p < 0.05: represents statistical difference between TGF-β free and TGF-β supplemented groups at corresponding time point. (**B-C**) Correlation curves between mechanical properties and biochemically-measured GAG and collagen contents for combination of both TGF-β supplemented and TGF-β free data points. Dashed curves represent exponential fits.

Similar to ECM contents, TE construct mechanical properties increased over culture duration and increased further with TGF-β supplementation (Fig. 4A). Consistent with prior investigations [27], [30], TE construct mechanical properties strongly correlate with GAG and collagen contents (Fig. 4B–C), suggesting that ECM biochemical measures can serve as a faithful indicator of mechanical integrity of engineered cartilage tissues. We observed a nonlinear relationship between biochemical content and mechanical properties. It is well established that engineered cartilage’s Young’s modulus correlates positively with ECM deposition (particularly with GAG content at early time points). However, the precise mechanistic details of these correlations can be highly complex, depending on mechanical anisotropy, tension-compression nonlinearity, and osmotic swelling pressures, and, as such, can often manifest in nonlinear mechanical-biochemical correlations. As such, the ECM measurements obtained via fiber-optic Raman spectroscopy can potentially serve as a strong predictor of engineered cartilage mechanical integrity and performance upon implantation. It is important to consider that while it is well established that the mechanical properties of articular cartilage are predominantly dependent on its contents of GAG and collagen, properties can further be influenced by other factors, such as levels of minor matrix constituents (e.g. crosslinks [31], small proteoglycans [32] and secondary collagen molecules [33]). Levels of these minor constituents cannot be readily quantified by our current fiber-optic analytical system and may vary with different TE systems that utilize different cell sources and scaffold types. As such, we anticipate that a unique biochemical/mechanical properties correlation may need to be established for each scaffold-cell source TE system. These unique correlations can be easily developed in the future during the translational stages of cartilage TE work.

### 3.3 Spectral similarity metrics between constructs and native tissue

**Fig. 5:**
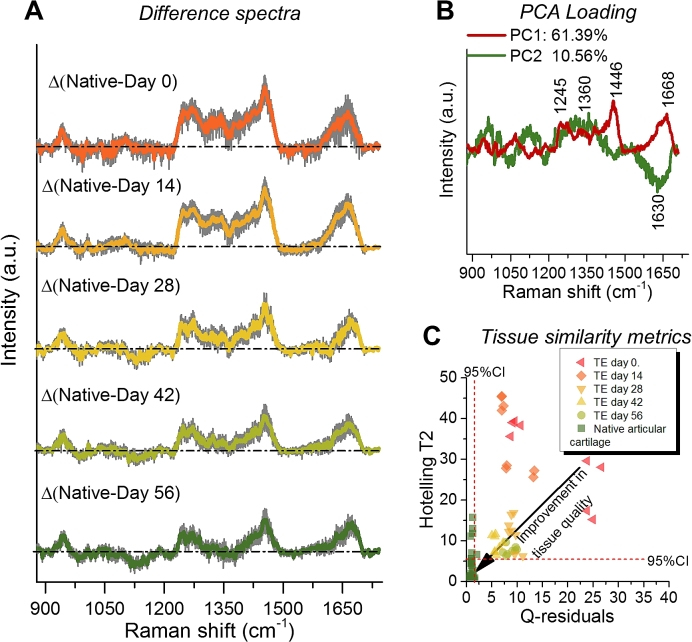
(**A**) Mean Raman difference spectra ±1 SD between native tissues and TE constructs at various time points (native – day 0, native – day 14, native – day 28, native – day 42, and native – day 56). (**B**) Principal component analysis (PCA) loadings developed from native articular cartilage. (**C**) Application of PCA model to TGF-β free TE constructs: Q-residuals plotted against the Hotelling T^2^ values for native articular cartilage tissues and TGF-β free TE constructs at day 0 (n = 8), day 14 (n = 8 spectra), day 28 (n = 8 spectra), day 42 (n = 8 spectra), day 56 (n = 8 spectra).

To better visualize the steadily changing biomolecular composition during tissue growth, we calculated the mean difference spectra ±1 SD between the various time points and native tissues (Fig. 5A). In an effort to quantify the similarity between TE constructs and native articular cartilage we developed a PCA model of two components based exclusively on the native tissue data (Fig. 5B). The PCA accounted for 71.95% of the total variance in the articular cartilage samples (PC1: 61.39% and PC2: 10.56%). Interestingly, the loading on PC1 was associated with proteins (*i.e.*, collagen) as indicated by peaks at 936, 1245, 1446 and 1668 cm^−1^. Loading PC2 showed peaks associated with more complex biomolecular variations in the tissue associated with GAG, water, and minor ECM components or chondrocytes (e.g., cytoplasm and DNA). These results show that the developed PCA model captures the high biomolecular complexity of native articular cartilage. We next projected the TE construct spectra onto this native model and calculated the Q-residuals and Hotelling T^2^ statistics (Fig. 5C). While the Q-residual and Hotelling T^2^ results indicate enormous biochemical differences between TE construct and native tissue it also shows that the overall biochemical composition of the TE constructs progressively resembles that of native tissue. The statistical multivariate analytical strategy developed here, therefore, provides a novel quantitative means to noninvasively evaluate our capability of replicating native tissues in terms of the overall biochemical composition. Since the developed framework only requires PCA modelling of the native tissue, which is readily obtainable, this approach can be applied to a host of other TE strategies for other scaffold materials and cell sources.

### 3.4 Online monitoring of live cell tissue engineered constructs

**Fig. 6:**
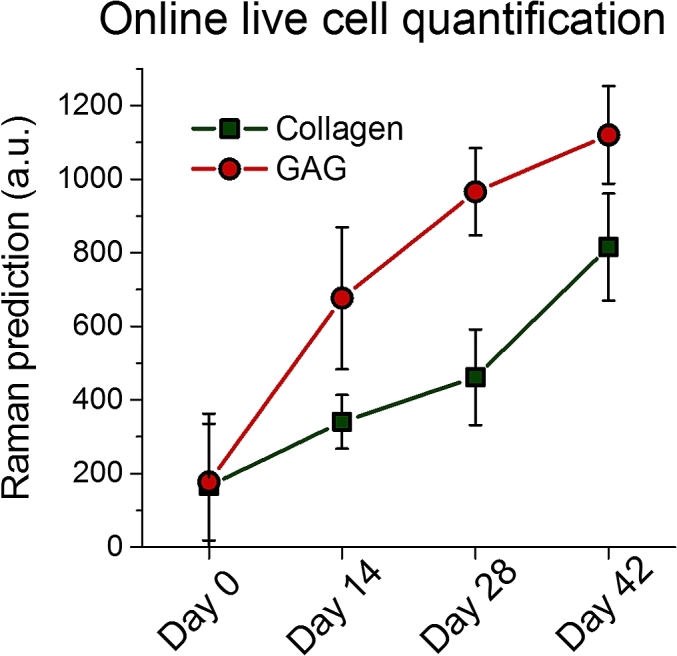
Online prospective application of the developed MCR model for online quantification of relative concentrations (arbitrary units) of collagen and GAG in live TE constructs at various time points (*i.e*. 0, 14, 28, and 42 days). Due to inherent difficulty in getting adequate signal to noise ratio at day 0 (since the majority of signal originates from water), only two data-points were measured.

To further demonstrate the application of our developed methodology we performed an additional online Raman spectroscopic analysis on live cell TE constructs. We measured Raman spectra of the live cell TE constructs at various time points throughout culture in a sterile environment. Fig. 6 shows the Raman spectroscopic measurement of relative GAG and collagen concentrations over 42 days of culture. This represents the first demonstration of monitoring and quantification of live cell TE constructs and shows a steady increase in collagen and GAGs during culture. Since we experienced fluctuations in fiber couplings throughput over time, we could not reliably convert these to absolute concentrations. Nevertheless, apart from an offset in prediction, these results further highlight the robustness of the developed technique for non-invasive biochemical analysis of TE constructs. In the future we aim to introduce an internal standard in the fiber design to thereby allow for absolute biochemical measurements over an extended time period.

Here we showed that fiber-optic Raman based quantification could provide information similar to that generated by standard experimental techniques such as biochemical assays of TE constructs but non-destructively and online during tissue culturing. In the future, the equipment and methodology can readily be expanded upon to allow for a more sophisticated non-invasive analysis of tissue growth. The application of spatial offset Raman spectroscopy (SORS) could potentially allow us to resolve the depth dependent zonal organization of the bulk TE constructs [34]. The quantification of tissue hydration was poor in this work due to inherently weak signals in the fingerprint spectral range (*i.e.* 800-1800 cm^−1^). Accurate quantification of tissue hydration can easily be implemented with high-wavenumber Raman spectroscopy in spectral range (*i.e.* 2800-3600 cm^−1^) [35].

We anticipate that this current analytical technique has broad applicability in the field of musculoskeletal tissue engineering. In principle, our fiber-optic Raman measurements are compatible with a wide range of tissue engineering systems, as most scaffold materials (e.g. hyaluronan, alginate, synthetic polymers) exhibit spectral signatures that are unique from the major cartilaginous ECM constituents (collagen and sulphated GAG). However, challenges could be encountered for scaffolds that exhibit significant spectral overlap with cartilage ECM (e.g. collagen or GAG incorporated scaffolds), necessitating the development of more sophisticated analytical models that can distinguish between exogenous and endogenous ECM constituents. Further, given the critical nature of ECM constitution for the robust mechanical functionality of most musculoskeletal tissues, fiber-optic Raman measurements can similarly serve as a critical translational quality control technique for tissue engineering of a large variety of tissues, including tendon, fibrocartilage, and bone.

The developed workflow will facilitate compilation of a large-scale tissue spectral database for monitoring a variety of TE constructs. It is particularly useful for the implantation of large pieces of tissue (e.g. full articular surface, entire meniscus, or a full tendon) where the sacrificing of additional samples to gauge ECM content is not a feasible option. We envision that this technique could be used as a tool for validation of TE construct maturation prior to implantation in patients as part of clinical therapies and serve as an efficient tool for regulatory approval of ECM formation in tissue. Future exciting applications of fiber-optic Raman spectroscopy in TE could combine the developed approach with other techniques, such as biomechanical testing or optical coherence tomography (OCT), to provide complimentary information on cell density and collagen alignment towards controlled clinical implantation of engineered constructs.

## 4 Conclusions

In summary, we have developed a fiber-optic Raman spectroscopy methodology for non-destructive quantitative biochemical analysis of the ECM in live cell TE constructs. A computational framework was developed to facilitate online quantitative analysis. We developed multivariate similarity metrics for comparison between TE constructs and native articular cartilage. Further, we devised a strategy that enables biochemical quantification of collagen and GAG in TE constructs with excellent correlation to biochemical assays. This study highlights the potential of diffuse fiber-optic Raman spectroscopy as a novel tool for characterization of native tissue and tissue-engineered constructs, allowing for non-invasive tissue analysis. The methodology developed here can be applied for ECM biochemical quantification in a number of tissue engineering applications.

## Conflict of interest

These authors declare no conflict of interest.

## Author contributions

M.S.B. and M.B.A designed the study, interpreted the data, and wrote the paper. M.S.B. conducted Raman spectroscopy work and data analyses. M.B.A cultured tissue-engineered constructs and performed biochemical assays. M.M.S. supervised the study, and contributed to the scientific discussions, data interpretation, and to the manuscript.
